# Sleep and obesity—known interactions and open questions

**DOI:** 10.1007/s44470-026-00130-7

**Published:** 2026-08-03

**Authors:** Christopher N. Schmickl, Rahul Gomez, Bernie Y. Sunwoo, Omar Mesarwi, Avnee J. Kumar, Atul Malhotra

**Affiliations:** 1 Division of Pulmonary, Critical Care, Sleep Medicine & Physiology, San Diego (UCSD), University of California, San Diego, La Jolla, CA 92093 USA; 2 Division of Pulmonary and Critical Care, Department of Veterans Affairs Medical Center, Jennifer Moreno, San Diego, CA USA

**Keywords:** Obesity, Insomnia, Sleep deprivation, Obstructive sleep apnea, Obesity hypoventilation syndrome

## Abstract

Sleep and obesity are tightly interconnected, with bidirectional relationships that influence cardiometabolic health and the development and progression of sleep disorders. In this review, we synthesize current evidence linking insufficient sleep, insomnia, obstructive sleep apnea (OSA), and other sleep disorders with obesity, with a focus on pathophysiology, clinical implications, and emerging therapeutic strategies. Insufficient sleep is consistently associated with weight gain through hormonal, behavioral, and metabolic mechanisms, whereas the relationship between insomnia and obesity appears weaker and more heterogeneous. Obesity is a major driver of OSA via effects on upper airway anatomy, respiratory mechanics, and ventilatory control, though reciprocal effects of OSA on weight remain less clear. Recent advances in obesity pharmacotherapy, particularly incretin-based therapies such as dual GIP/GLP-1 receptor agonists, will likely reshape treatment paradigms. Clinical trial data demonstrate meaningful improvements in both weight and OSA severity, highlighting the potential for integrated management of comorbid obesity and OSA (COBOSA); however, traditional therapies—including continuous positive airway pressure (CPAP), exercise, and lifestyle modification—remain foundational components of care. We also review obesity hypoventilation syndrome and other sleep disorders associated with obesity, emphasizing shared and distinct mechanisms. Despite substantial progress, key knowledge gaps remain regarding optimal treatment integration, heterogeneity of treatment response, and long-term outcomes. Overall, effective management of obesity is increasingly central to sleep medicine, underscoring the need for multidisciplinary, patient-centered approaches and further research. Likewise, promoting adequate sleep should be considered an important component of weight management and overall health.

The three pillars of health are sometimes referred to as diet, exercise, and sleep with the dogma being that if you ignore one, the other two will suffer [1, 2]. People who are sleep deprived tend to limit daily activity and eat excessively, often non-nutritious foods [3, 4]. People who do not exercise do not sleep well and often gain weight, increasing their risk of obstructive sleep apnea (OSA) [5–7]. Some data support the notion that exercise itself may protect against sleep apnea independent of body weight [6]. These bidirectional relationships between poor sleep, weight gain, and insufficient exercise often compound each other and pose major challenges for patients and their healthcare providers. In this review, we explore current thinking regarding the links between sleep issues and obesity in adults with an emphasis on therapeutic interventions and unanswered questions.

## Obesity

**Fig. 1:**
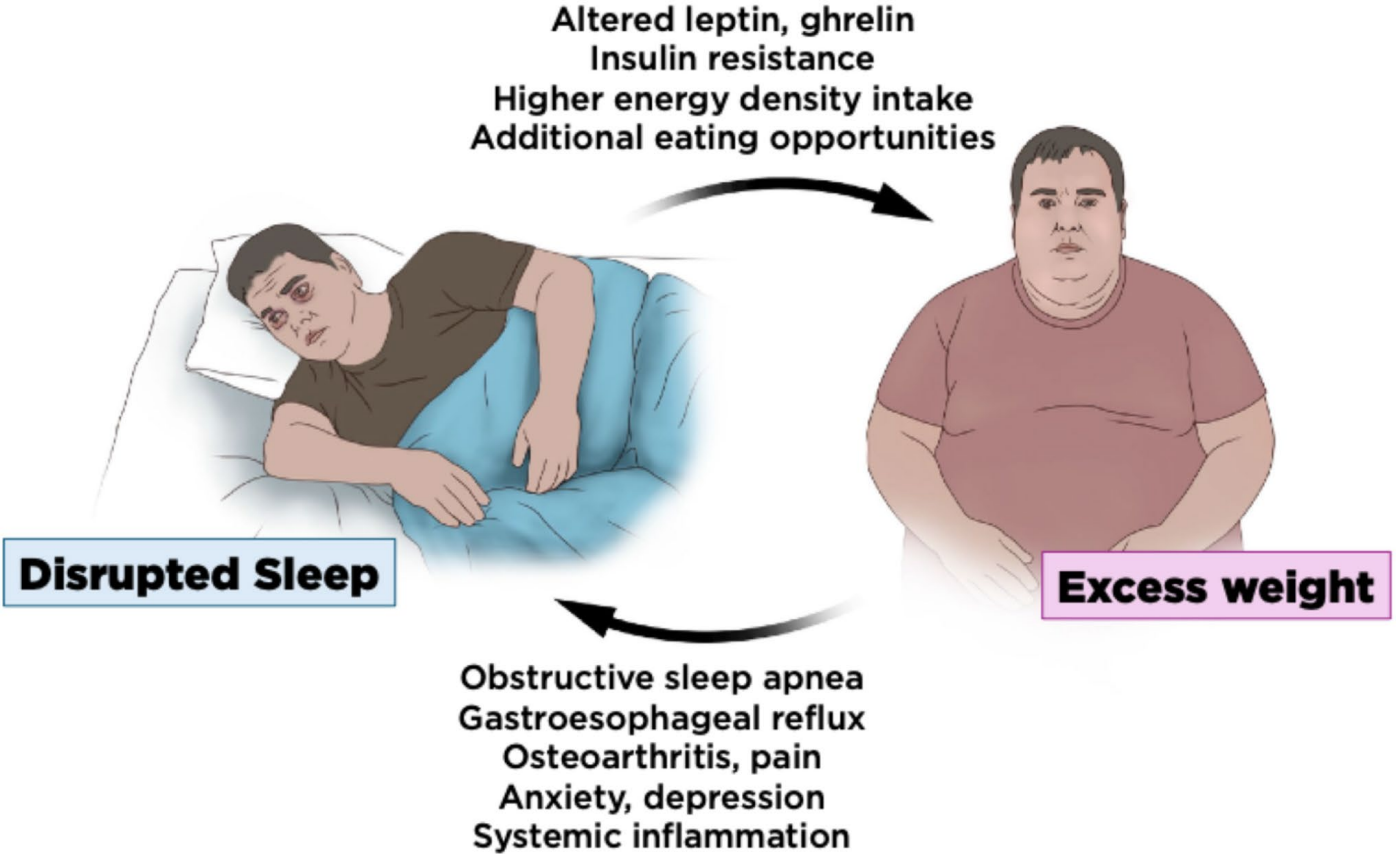
Mechanisms which are known to account for the bidirectional relationship between excess weight and sleep disruption

Obesity is defined as excess body fat, typically diagnosed by a body mass index (BMI) ≥ 30 kg/m^2^ (≥ 27.5 kg/m^2^ in Asian populations) [8], and affects over 40% of US adults with disproportionately high prevalence among certain racial, ethnic, and socioeconomic subgroups (e.g., individuals reporting non-Hispanic Black race and those with low income) [9, 10]. Although widely used, BMI is an imperfect measure of excess adiposity and in individuals with BMI 30–40 kg/m^2^ should be interpreted in the context of additional anthropometric measures [8]. Given its high prevalence, obesity commonly coexists with sleep disorders and, as discussed below, frequently interacts with them (Fig. 1).

While obesity increases the risk for over 200 diseases (diabetes, heart disease, cancer, OSA, etc.), it is now widely recognized as a chronic disease in its own right [11]. The recent Lancet Diabetes and Endocrinology Commission placed an emphasis on differentiating clinical obesity from preclinical obesity, based on the presence or absence of reduced organ or tissue function due to obesity and/or daily activity limitations [8]. The pathophysiology of obesity is complex, driven by interactions between genetic, neurohormonal, environmental, and behavioral factors—with the energy homeostasis system actively defending an elevated fat mass “set point” [12]. Caloric restriction does not change this set point but triggers persistent hunger while reducing energy expenditure [8, 12], explaining the commonly observed weight regain with diet alone [13–15]. Thus, even in comprehensive lifestyle programs—access to which is often limited—effects are typically temporary and modest (2–5% decrease in weight) [13–16]. Earlier pharmacotherapies (orlistat, phentermine-topiramate, and naltrexone-bupropion) had limited effectiveness (5–10% decrease in weight) and tolerance [17–19]. Bariatric surgery is highly effective (often > 20–25% weight loss) [20–22] but invasive and generally reserved for severe disease.

Recognition that gut-derived nutrient-stimulated hormones (NuSH)—particularly glucagon-like peptide 1 (GLP-1) and glucose-dependent insulinotropic peptide (GIP)—regulate appetite, satiety, and energy balance has led to newer treatments for obesity [23]. Incretin-based therapies, including dual GIP/GLP-1 receptor agonists such as tirzepatide, lower the defended fat mass set point, producing substantial and sustained weight loss and reframing obesity as a biologically treatable condition [24]. These advances in incretin- and broader NuSH-based therapies have important implications across specialties [25], including sleep medicine, where obesity is a driver and/or consequence of several disorders. Important questions remain regarding how best to integrate these therapies into existing treatment pathways in an equitable manner. There is emerging evidence for heterogeneous treatment effects of GLP1 receptor agonists (e.g., less weight loss in individuals with diabetes [16, 26], while women tend to lose more weight than men [27]), but the mechanisms and clinical implications of these differential responses are unclear.

## Insufficient sleep syndrome

Approximately one third of US adults obtain less than the recommended minimum of 7 h of sleep per night, with particularly high prevalence among younger men, individuals of lower socioeconomic status, rural residents, and non-Hispanic Black adults [28, 29]. Insufficient sleep has been consistently associated with higher body weight in cross-sectional studies [30, 31] and experimental data provide mechanistic support for a causal contribution to positive energy balance. In elegant studies by Spiegel, Tasali and Van Cauter, the authors have observed hormonal changes which occur in the context of sleep deprivation [4, 32]. For example, in one randomized trial, short sleepers experienced reductions in leptin (a satiety hormone) and increases in ghrelin (an appetite-stimulating hormone), both hormones going in directions that would be predicted to stimulate appetite [4]. Of note, the ratio of leptin to ghrelin increased fairly linearly with sleep deprivation as emphasized in a prominent editorial by Flier [33]. Individuals who experience sleep deprivation frequently report craving non-nutritious foods [34].

These findings were later corroborated by epidemiological data by Patel et al. who analyzed data from the Nurses’ Health Study [35]. The participants were asked about typical sleep duration and followed prospectively over time. The nurses reporting 5 h per night of sleep weighed more at baseline than those reporting 7–8 h and gained more weight over time compared to those sleeping adequately. The large sample size of 68,183 women allowed controlling for known covariates, and the adjusted results supported the notion that sleep deprivation may be an independent risk factor for incident obesity. Subsequently, a randomized study examined dietary interventions (reduced energy intake) and found that sleep deprivation led to substantial impairment in diet-induced improvement in fat free mass [36]. Similarly, in another trial subjects limited to 4 h of sleep per night, consumed on average an additional 308 kcal per day compared to those in the control group with a sleep opportunity of 9 h [37]. This same trial showed a weight gain on average of 0.5 kg over the control group, and in particular showed increases in visceral fat, which has been closely associated with cardiometabolic risk. Conversely, a recent trial found that sleep extension by 1.2 h led to reduced energy intake and a negative energy balance in a real-world setting among adults with sleep deprivation [38].

Sleep deprivation has also been shown to impair exercise performance [39], potentially mediated in part by increases in inflammatory cytokines such as IL-6 [40]. In aggregate, these data suggest that addressing insufficient sleep may support weight regulation, enhance athletic performance, and improve metabolic health, with potential benefits for reducing sleep-related health disparities.

## Insomnia

Insomnia is highly prevalent, affecting over 10% of adults in the USA [41]. Despite strong evidence linking short sleep duration with obesity, the relationship between insomnia and adiposity is less clear. In principle, a subgroup of insomnia characterized by objectively short sleep duration may share mechanistic pathways with insufficient sleep, including altered appetite regulation and reduced energy expenditure. However, a meta-analysis of predominantly cross-sectional studies found no significant association between insomnia and obesity status; when analyzing insomnia symptoms and BMI, the same authors found a small effect (*r* = 0.06; *p* = 0.03) but heterogeneity was high (*I*^2^ = 99%) [42]. Furthermore, meta-analyses comparing insomnia with short sleep duration versus insomnia with normal sleep duration did not reveal an increased obesity risk [43]. A recent study of cognitive behavioral therapy for insomnia (CBTI) in adults with short (< 7 h) and disturbed sleep noted improvements in food preferences, but no changes in self-reported food intake [44]. Effects on BMI are generally not assessed in CBTI studies [45]. Thus, based on current evidence from mostly cross-sectional studies, insomnia does not appear to increase the risk of obesity. One potential explanation is that physiological hyperarousal, a central feature of insomnia, may offset obesity-promoting mechanisms [46]. However, longitudinal and more mechanistic/interventional studies are needed to clarify this relationship.

Evidence for the reverse relationship is similarly limited. Although obesity was a predictor of incident insomnia in one prospective cohort study [47], a meta-analysis including data from two additional longitudinal cohorts did not find a significant relationship (average follow-up 8.2 years) [42].

Collectively, these findings indicate that, unlike short sleep duration, insomnia appears to have a weak relationship with obesity, potentially reflecting residual confounding and challenges distinguishing insomnia from comorbid sleep disorders such as obstructive sleep apnea which often coexists and sometimes may even mimic insomnia [48]. An important related entity is comorbid insomnia and sleep apnea (COMISA), which is associated with greater symptom burden, worse CPAP adherence, and poorer cardiometabolic outcomes than either condition alone [49, 50]. In patients with COMISA, CBT-I may improve insomnia symptoms and facilitate subsequent CPAP acceptance and adherence [51]. Given these observations, future studies should also examine whether early treatment of obesity, when present in these patients, may similarly improve long-term treatment engagement and outcomes.

## Obstructive sleep apnea

Obstructive sleep apnea (OSA) is estimated to affect up to 1 billion people worldwide, with recent modeling studies projecting that these numbers will continue to increase over time, particularly in women [25, 52, 53]. The reason for the rising prevalence is related to the aging of the population and the obesity pandemic which continues despite the impact of new obesity medications [54]. The term COBOSA (comorbid obesity and OSA) has recently been coined to emphasize the frequent co-occurrence of these conditions and—given the advent of potent weight-loss medications—to highlight a clinically important subgroup of patients with OSA [55]. Approximately half of OSA cases are attributable to excess body fat, [56, 57] representing a substantial population in whom weight reduction should be a central part of the treatment strategy as it promises broad health benefits beyond improvements in sleep-disordered breathing [24, 58]. However, weight loss requires time (typically ~ 1 year before weight plateaus with both bariatric surgery and incretin-based pharmacotherapy) [15, 22], its effect on OSA severity on an individual level is variable [16, 59], and a substantial fraction of patients have sleep apnea in the absence of obesity—especially in community cohorts [60]. Moreover, addressing obesity and OSA concurrently may yield synergistic benefits [61, 62], and OSA treatment likely improves outcomes across multiple domains [63], underscoring the need to address OSA as a major public health problem in patients with and without comorbid obesity [64].

### Pathophysiological links between obesity and OSA

OSA pathogenesis is now understood to be complex, with general acknowledgment that not all OSA patients get their disease for the same reason [65]. Instead, there are multiple pathophysiological mechanisms underlying OSA including anatomical compromise, upper airway dilator muscle dysfunction, unstable control of breathing (also known as high loop gain), low arousal threshold, reduced end-expiratory lung volume and other factors [66].

Obesity influences several of these pathways. Fat deposition in the tongue and the parapharyngeal tissues can alter pharyngeal anatomy [67, 68] and lead to increased pharyngeal collapsibility, with rigorous studies showing improvement in collapsibility following weight loss [69–71]. Additionally, abdominal fat can contribute to reduced end-expiratory lung volume which reduces longitudinal tethering on the upper airway promoting pharyngeal collapse [72–74]. Thus, the distribution of body fat mass may be critical regarding impacts to pharyngeal mechanics. Regarding upper airway dilator muscle function, Sands et al. observed that people with obesity without OSA had robust upper airway “gain” as compared to matched individuals with obesity and OSA [75]. Thus, upper airway muscles may be sufficient to prevent pharyngeal collapse and could represent a therapeutic target in this context. Unstable control of breathing can also be impacted by obesity [76–79]. Loop gain would be predicted to be elevated with reduced lung stores (so-called plant gain), baseline hypoxemia (which could stimulate ventilation), and with variable upper airway dynamics (i.e., component of the controller gain). Obesity may also contribute to OSA by shortening circulatory delay (i.e., circulation time between the lung and chemoreceptors; “mixing gain”) through an increase in cardiac output, which may lead to shorter but more frequent respiratory events [80]. Mediation analyses further suggest sex-specific pathways linking obesity to OSA, with upper airway collapsibility accounting for a relatively greater proportion of obesity-related risk in men than in women [80]. Collectively, these findings demonstrate that the contribution of obesity to OSA pathogenesis is complex and likely varies across patients. This endotypic heterogeneity has important implications for weight loss response: patients with predominantly anatomically-driven OSA (high collapsibility, large parapharyngeal fat deposits) are likely to respond most robustly to weight reduction. In contrast, those with non-anatomical drivers such as low arousal threshold may have a more modest AHI response to weight loss, even when substantial. Whether tirzepatide itself influences non-anatomical endotype traits via direct neural GLP-1 receptor effects remains unknown and represents an important research question.

Of note, several mechanisms have been proposed whereby OSA may contribute to obesity, including via alterations in appetite-regulating hormones and reduced physical activity due to sleepiness [81]. However, meta-analyses of interventional studies evaluating OSA treatment showed overall slight weight gain rather than weight loss (see below for potential explanations) [82]. Thus, while obesity is a well-established driver of OSA, potential reciprocal effects of OSA on obesity are less clear.

### Key components in the management of COBOSA

*Nasal CPAP* is the first-line therapy for many patients with OSA, particularly those with moderate-to-severe disease, and can have transformative benefits for some patients [83, 84]. Rigorous data support that CPAP can lead to improvements in symptoms as well as blood pressure while suggestive data support the notion that CPAP can reduce cardiovascular risk [64]. In the context of this review, CPAP has been associated with small but significant increases in body weight, although underlying mechanisms are unclear [82]. Several theories have been proposed. First, some studies found that the CPAP-induced weight gain reflects an increase in fat-free mass, potentially representing an increase in muscle mass and thus improved body composition [85, 86]. Other studies have found increases in extracellular fluid volume, whereby the increase in fat-free mass may instead, or in part, reflect fluid accumulation [87, 88]. In either case, increases in lean mass may not necessarily indicate adverse metabolic effects. Second, hormonal theories have been suggested such that CPAP may induce changes in slow wave sleep which could contribute to release of growth hormone and other factors [89, 90]. Third, respiratory muscle work can be considerable in untreated OSA patients struggling to breathe. As such, CPAP may reduce respiratory work considerably by promoting upper airway patency [91, 92]. Thus, daytime activity may have to increase markedly to overcome the reduced caloric expenditure associated with improved breathing. Fourth, and perhaps the most likely, is that CPAP leads to personal and behavioral changes which involve caloric consumption. Many patients report “getting their life back” which can involve going for dinner with partner or beers with friends—social activities which involve caloric intake. At present, the mechanism for the small CPAP-induced weight gain is unclear but should be studied further [93], while physicians should monitor and address weight changes following CPAP initiation as appropriate. Obesity can also influence the effectiveness and candidacy of several non-PAP therapies. Oral appliance therapy (OAT) tends to be less effective in patients with higher BMI, while eligibility and outcomes for hypoglossal nerve stimulation (HNS) and sleep surgery have historically been more limited in patients with obesity [94, 95]. The potential neoadjuvant role of incretin-based pharmacotherapy to improve candidacy and treatment outcomes with HNS, OAT, or sleep surgery represents an intriguing area for future study [96, 97].

*Exercise* is frequently recommended for patients in the sleep clinic but more broadly for society [98]. Increasing energy expenditure promotes weight loss if caloric intake does not increase concomitantly, helps preserve muscle and bone mass during weight loss—especially when aerobic and resistance training are combined [99]—and strongly predicts weight loss maintenance [98, 100]. In clinical practice exercise goals are achieved inconsistently and thus weight loss from exercise can be quite variable. Some data support the notion that exercise itself can be helpful for OSA even without major changes in body weight. Recent meta-analyses showed that exercise training can significantly improve OSA severity, with reductions in apnea–hypopnea index (AHI) by 5.2 to 8.9 events per hour despite no improvement in BMI [101, 102]. Most studies focused on aerobic exercise, but resistance training alone appears also to be effective in improving OSA [103]. In contrast, physical activity related to occupation may be less beneficial based on observational data [104]. The mechanism whereby exercise improves sleep apnea is unclear but may partly relate to activation of upper airway muscles that occurs with concomitant activation of the diaphragm during aerobic activity [105]. In observational studies, confounding by socioeconomic status and/or reverse causation may be important whereby patients with symptomatic OSA may be less active than those without OSA. Notably, some data suggest that treatment of OSA may improve functional exercise capacity (VO_2_ peak) [106], which could facilitate greater physical activity and promote a “virtuous cycle”. In aggregate, based on current evidence and clinical experience, exercise should be recommended for most patients to help improve sleep and promote overall health.

**Fig. 2:**
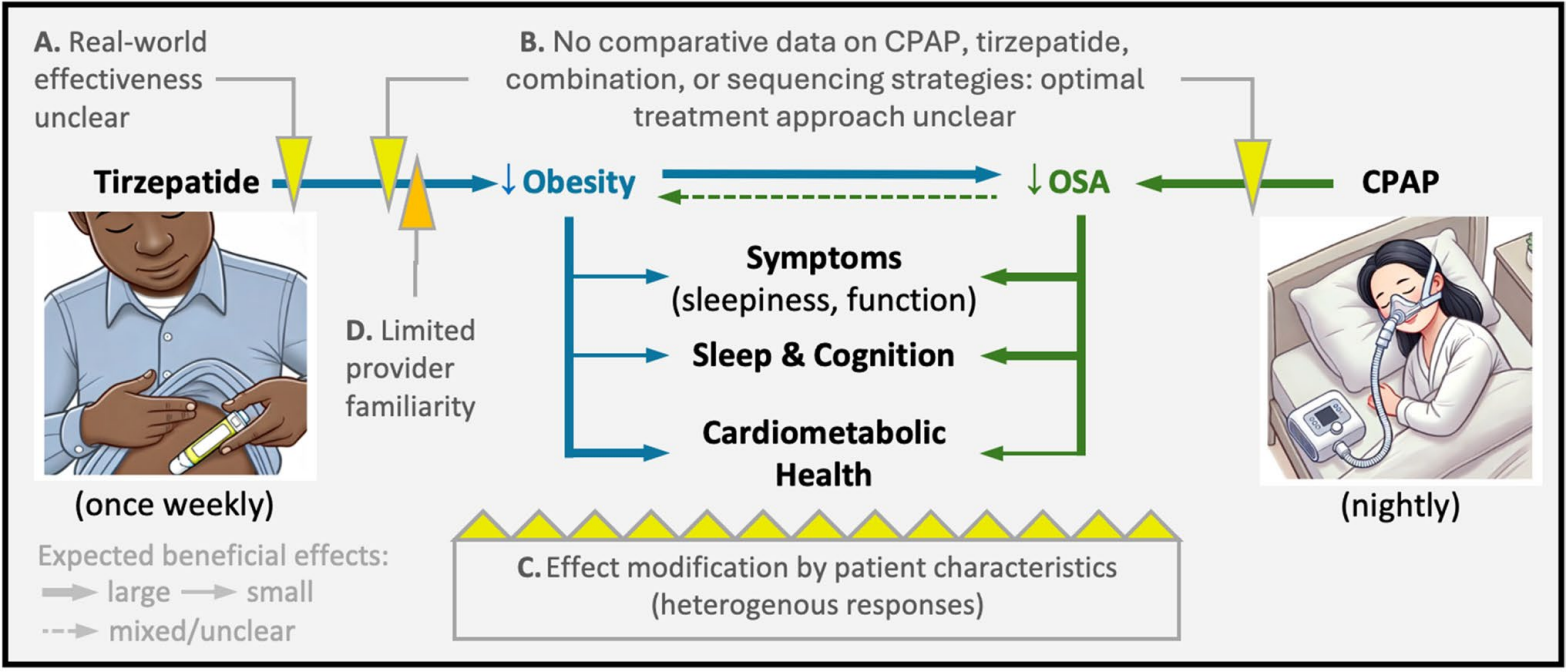
Critical knowledge gaps (yellow) and barriers (orange) for the management of patients with comorbid obesity and OSA (COBOSA). Modified from [110]
- Are the findings unique to tirzepatide or would the same results be achievable with other incretin- or NuSH-based weight loss strategies? Prior studies with liraglutide suggest that, in the small proportion of patients achieving similar percentage weight loss, the effect on OSA was comparable to more potent agents [112]. Diet and lifestyle interventions have also demonstrated OSA improvements proportional to weight loss achieved [22, 107, 113–115], suggesting weight loss—rather than the specific pharmacological mechanism—as the primary driver. Next-generation triple agonists (e.g., retatrutide) achieving even greater weight loss will further test this hypothesis [116]. The longevity of clinical efficacy also warrants consideration: bariatric surgery provides durable weight loss over a decade or more for most patients [20–22], but revision surgeries are often required over time [21]. Pharmacotherapy requires ongoing adherence [117, 118], in which case current data show that weight loss with GLP-1-RAs can be maintained for at least 3–4 years [24, 119, 120]. However, recent real-world studies of mostly older GLP-1 agents report frequent discontinuations within one year [121, 122], in part likely due to limited access and coverage. Notably, one study found that greater weight-loss efficacy improves adherence [122], supporting optimism for potent GLP-1 s, especially once lower-cost options become available—with generic agents expected within 5–7 years in many jurisdictions. In parallel, expanding availability of obesity pharmacotherapy may further increase the importance of obesity-management competencies within sleep medicine practice.
- Will tirzepatide work in patients not included in SURMOUNT-OSA? For example, people with diabetes, mild sleep apnea, lean or overweight without obesity, and others were not included in SURMOUNT-OSA, so additional studies will be required to determine the generalizability of the findings.
- How to address residual symptoms? As tirzepatide and related agents become more widely used, clinicians will increasingly encounter patients who have lost substantial weight, whose CPAP data appear satisfactory, yet who remain significantly sleepy. This “residual sleepiness in treated, weight-reduced COBOSA” is an emerging clinical phenotype with a wide differential: residual or recurrent OSA despite weight loss, an unmasked central hypersomnolence disorder (e.g., narcolepsy type 2 or idiopathic hypersomnia previously attributed to OSA), medication effect, mood disorder, or chronic insufficient sleep. Prospective work characterizing this phenotype is needed.
- Was the mechanism of improvement entirely attributable to weight loss? A recent mediation analysis was published in Nature Medicine which examined the relative impact of weight loss vs. OSA improvement vs. direct effects of tirzepatide [62]. The results showed variable effects for different outcomes of interest. For example, blood pressure improvements were largely attributable to weight loss rather than AHI improvement per se. On the other hand, for hsCRP and for insulin resistance, there were important effects of both weight loss and AHI improvement on the observed outcomes.
- Does clinically relevant sarcopenia develop with pharmacologic weight loss, and could this influence sleep-disordered breathing outcomes? Some loss of lean mass is predictable after major weight loss, with phase 3 incretin-based obesity trials suggesting roughly 20–40% of weight lost is from lean mass rather than fat mass [123]. While reductions in visceral and upper-airway adiposity are generally expected to improve OSA, substantial loss of pharyngeal dilator, respiratory, or other skeletal muscle could theoretically impair upper-airway stability or ventilatory reserve, particularly in vulnerable populations such as patients with obesity hypoventilation syndrome (OHS). At present, the clinical significance of these changes remains unclear. Resistance exercise and adequate protein intake currently represent the best-supported strategies to preserve lean mass during weight loss [99, 124]. In parallel, emerging pharmacologic approaches targeting muscle preservation—including androgen modulation, and especially myostatin/activin pathway inhibitors such as bimagrumab [123, 125, 126]—may help mitigate weight-loss-associated muscle loss and warrant further investigation in patients with sleep-disordered breathing. Future studies should examine not only weight loss but also body composition, respiratory muscle function, and upper-airway physiology.

*Weight loss* was shown to improve OSA in a recent meta-analysis, whether achieved by lifestyle interventions, bariatric surgery or pharmacologically [107]. The recent SURMOUNT-OSA study has led to a paradigm shift in OSA management. In two randomized studies, one arm not on CPAP and one arm using CPAP, tirzepatide (a dual GIP and GLP-1 receptor agonist) led to clinically and statistically significant improvements in the AHI compared to placebo [15]. In addition, a number of pre-specified secondary outcomes were controlled for multiple comparisons and showed major improvements including systolic blood pressure, high-sensitivity C-reactive protein (CRP), sleep apnea-specific hypoxic burden, patient-reported outcomes, and body weight. These data led to FDA approval of tirzepatide for treatment of moderate to severe OSA in people with obesity (although, at the time of writing, regulatory approval for OSA varies internationally). Although clinical uptake may be gradual—reflecting limited familiarity among sleep clinicians [108], uncertainties regarding implementation, and insurance barriers—the emergence of incretin- and NuSH-based therapies represents a shift toward greater integration of obesity pharmacotherapy into sleep medicine practice. Analogous to the central role of statins in cardiovascular care, effective management of obesity is likely to become an essential component of comprehensive OSA treatment, underscoring the need for sleep clinicians to develop expertise in the use of these therapies in collaboration with obesity and primary care specialists. However, a number of questions remain following SURMOUNT-OSA:

- How to best integrate tirzepatide in the care of patients with COBOSA (Fig. 2). How effective is tirzepatide outside tightly controlled trial settings—which included intensive lifestyle counseling and adherence monitoring—and implemented in real-world sleep clinic practice? How does its effectiveness compare with CPAP [109, 110], and are there additive/synergistic benefits when the two therapies are combined? Of note, many patients would prefer tirzepatide over CPAP if both were similarly effective and find combination therapy acceptable despite increased patient and healthcare system burden if it provided superior outcomes relative to either treatment alone [108]. Beyond the question of which therapy to initiate, important unanswered questions include how best to sequence, combine, escalate, or de-escalate therapies over time under real-world conditions. For example, some patients may benefit from early combination therapy followed by later CPAP discontinuation after substantial weight loss, whereas others may require addition of CPAP, HNS, or other therapies if weight loss alone proves insufficient. Better understanding of these longitudinal treatment pathways and heterogeneous treatment responses will likely be critical to individualized care, particularly as many sleep providers currently have limited familiarity with obesity pharmacotherapy and integrated obesity management [111].

## Obesity hypoventilation syndrome

The most severe form of obesity-induced respiratory compromise is obesity hypoventilation syndrome (OHS). Defined by the presence of obesity, daytime hypercapnia during wakefulness (PaCO_2_ ≥ 45 mm Hg) in the absence of other causes for hypoventilation and in the setting of sleep-disordered breathing (most commonly OSA), OHS is associated with worse outcomes including mortality, increased healthcare utilization, heart failure, pulmonary hypertension and lower quality of life than eucapnic patients with OSA [127–131]. While there has been ongoing debate on whether OHS represents the most severe end of a continuum of sleep-disordered breathing versus a distinct entity, the risk of OHS has clearly been associated with severity of obesity [132–135]. An estimated 10–17% of patients with OSA have OHS but, in individuals with a BMI > 50 kg/m^2^, prevalence as high as 50% has been reported [132, 136–138]. Why and how a subset of individuals with obesity go on to develop daytime hypercapnia remains incompletely understood. Like OSA, the mechanism of OHS is felt to be likely multifactorial including but not limited to changes in respiratory mechanics, impaired central ventilatory drive, sleep-disordered breathing and excessive CO_2_ production.

Obesity, and in particular central obesity, imposes a mechanical load on the respiratory system, further exacerbated in the supine body position with cephalic displacement of the diaphragm. Lung and respiratory system compliance is reduced. Obese individuals breathe at low lung volumes, and at these low lung volumes, there are small airway closure and expiratory flow limitation leading to increased work of breathing and disturbed ventilation-perfusion relationships. Normally, the increased workload is offset by an increase in respiratory drive, but in OHS this drive is blunted. OHS has been associated with a reduction of ventilatory responsiveness to hypoxia and hypercapnia [139, 140]. Leptin resistance may be one mechanism for this reduced central respiratory drive. Leptin is a protein encoded by the ob gene that acts on the long isoform of leptin receptor (LEPR^b^) in the brain to produce satiety, stimulate ventilation and improve upper airway patency during sleep [141, 142]. In mice genetically deficient in leptin, there is a blunted ventilatory response compared to wild-type mice. Leptin resistance is attributed to limited permeability of the blood–brain barrier to leptin and impaired leptin signaling and there is growing interest in leptin and its signaling pathways as potential therapeutic targets for OHS [143]. In approximately 90% of patients with OHS, the sleep-disordered breathing seen is OSA with 70% having severe OSA. The risk of developing daytime hypercapnia increases with OSA severity and is thought to result from inadequate compensation for nocturnal hypercapnia by either the lung or kidney. Typically, obstructive apneas and hypopneas result in a transient increase in CO_2_, but obstructive respiratory events are usually followed by arousals and augmented ventilation to eliminate the accumulated CO_2_ to maintain a steady-state arterial PaCO_2_. Any imbalance between the duration of obstructive respiratory events and compensatory inter-apnea periods may lead to net CO_2_ accumulation, bicarbonate elevation, and eventual daytime hypercapnia [144–146]. Finally, obesity may contribute to the development of hypercapnia through CO_2_ hyperproduction. Javaheri et al. examined respiratory variables in eucapnic and hypercapnic patients with OSA matched for AHI to determine mechanisms of diurnal hypercapnia [147]. While they hypothesized OHS patients to have lower minute and alveolar ventilation than eucapnic OSA patients, they found similar ventilation between the two groups but greater CO_2_ production in the OHS group that was not significant when adjusted for body surface area, again highlighting the role of weight in OHS.

Further supporting the role of obesity in OHS is improvement in hypercapnia and hypoxemia, daytime sleepiness, AHI, pulmonary function and cardiovascular and metabolic outcomes shown with weight loss. However, similar to OSA, the degree of weight loss necessary to achieve clinically significant improvement is unclear. This target weight loss is likely to vary depending on the chosen outcome(s) in OHS [148]. Most studies on OHS focus on resolution of hypercapnia as the target outcome but the degree of weight loss needed to improve hypercapnia may not be the same as the degree of weight loss needed to improve other outcomes like cardiometabolic complications. In 2019, the American Thoracic Society (ATS) made a conditional recommendation suggesting weight-loss interventions that produce sustained weight loss of 25–30% of actual body weight in OHS [149, 150]. Although based on very low-quality evidence, this extent of weight loss was felt most likely to achieve resolution or clinically meaningful reduction of hypoventilation, with again greater weight loss associated with better outcomes [151]. Notably, this 25–30% weight-loss target has historically required bariatric surgery, and real-world bariatric data confirm its feasibility and impact on OHS [150]. Emerging incretin- and broader NuSH-based pharmacotherapies, including dual and triple agonists, may allow some patients to achieve comparable weight loss non-surgically, potentially reshaping treatment paradigms in OHS. Whether substantial pharmacologic weight loss can reduce long-term dependence on ventilatory support, alter the relative roles of CPAP versus NIV [152, 153], or modify disease trajectory in OHS remains unknown and warrants prospective study. In parallel, therapies with established cardiometabolic benefits, such as SGLT-2 inhibitors, may also merit further study in obesity-related sleep-disordered breathing given their favorable effects on visceral adiposity, heart failure outcomes, and potentially nocturnal fluid redistribution [154].

## Other sleep disorders associated with obesity

### Hypersomnolence

Several additional sleep disorders demonstrate associations with obesity. Narcolepsy type 1 (NT1, orexin-deficient) is consistently linked to weight gain and increased adiposity, often emerging near disease onset [155, 156]. The mechanisms underlying this weight gain are incompletely understood but may include orexin-deficiency-related reductions in energy expenditure and alterations in food-seeking behavior [155, 156]. In contrast, the relationship between narcolepsy type 2 (NT2, normal CSF orexin levels), orexin signaling, and obesity is less well defined and may differ substantially from NT1, highlighting the importance of not conflating these entities. NT2 patients are also frequently overweight or obese, and whether orexin agonism might favorably affect body composition in this group is an important unanswered question. Treatment of patients with NT1 with sodium oxybate and stimulants generally results in modest weight loss [156]. Emerging orexin agonists promise a precision-medicine approach to NT1 that restores near-normal levels of alertness and night-time sleep [157]. While early reports do not suggest major improvements in body weight with orexin agonists, these agents may provide important mechanistic insights into the links between arousal regulation, metabolism, and obesity. More broadly, growing pharmaceutical investment in both obesity therapeutics and orexin-based sleep/wake therapeutics underscores the increasingly recognized overlap between sleep regulation, energy homeostasis, and cardiometabolic disease, and may have important implications for future trial design and integrated treatment pathways. Additionally, hypersomnolence itself appears associated with obesity independent of OSA, [158–161] further supporting links between arousal regulation, metabolism, and energy balance. Night eating syndrome and sleep-related eating disorder also deserve consideration in patients with obesity presenting with sleep complaints.

### Genetic syndromes

Genetic syndromes such as Prader–Willi syndrome represent extreme examples of combined sleep and metabolic dysregulation, characterized by severe obesity and frequent sleep-disordered breathing. Careful studies of these patients may provide insight into shared pathways and inform novel therapeutic strategies for obesity and OSA [162, 163]. Individuals with Down syndrome also exhibit elevated rates of obesity, potentially driven by leptin resistance, reduced resting energy expenditure, unfavorable dietary patterns, and comorbidities (e.g., hypotonia and micrognathia). Obesity likely contributes to the high prevalence of OSA in this population and represents a potential therapeutic target for sleep providers. However, non-anatomical factors (e.g., hypotonia of upper airway dilators) may also play an important role in OSA pathogenesis, underscoring the need for rigorous evaluation of tirzepatide or similar weight-loss medications in individuals with Down syndrome.

### Other sleep disorders

Finally, circadian rhythm sleep–wake disorders, particularly shift work, are associated with increased risk of obesity—especially abdominal adiposity—likely mediated by eating during the biological night and insufficient sleep resulting from circadian misalignment and daytime sleep disruption [164]. Strategies such as rotating schedules and optimizing the daytime sleep environment may help mitigate these risks [164], although evidence guiding interventions for obesity prevention and treatment in the context of circadian disorders remains limited.

Across these diverse conditions, several mechanistic themes recur. Leptin signaling appears important in regulating both ventilatory drive and satiety, with potential relevance across OHS, Prader–Willi syndrome, and Down syndrome. Orexinergic signaling modulates arousal, food-seeking behavior, and energy expenditure, linking narcolepsy—particularly type 1—with metabolic dysregulation. In parallel, circadian disruption can impair both sleep quality and metabolic homeostasis, helping explain the association between shift work and obesity risk. Recognizing these shared pathways underscores the increasingly interconnected nature of sleep and metabolic medicine and may help inform future mechanistically targeted therapies.

## Future directions

Despite considerable progress in our understanding of the interplay between sleep and obesity, a number of important knowledge gaps remain. In addition to the open questions highlighted throughout this review, we suggest the following key areas warranting further investigation:

1. *Long sleep* has been associated with excess body weight and with weight gain over time. The mechanisms underlying this observation are unclear but require further mechanistic study. Epidemiological studies have shown a loose association between long sleep and inflammatory conditions as well as depression but these factors seem insufficient to explain the very consistent observation that long sleep leads to poor health outcomes. Our clinical experience is that habitually long sleepers are often socially isolated without family support or children and thus perhaps psychosocial factors play an important role. Nonetheless, we are strongly supportive of mechanistic and interventional studies to address this unanswered question.
2. *Public health awareness*: Although progress is ongoing, there is considerable room for improvement regarding public awareness regarding the importance of sleep and sleep health. In many circles, statements such as “he never sleeps” are regarded as a compliment regarding an individual’s work ethic rather than a strong marker of cardiometabolic risk. Similarly, in some cultures, saying “he snored all night” is regarded as a marker of good sleep rather than a harbinger of desaturations and catecholamine surges from OSA. Of note, the American Heart Association recently added sleep to the Essential Eight, which has helped to raise awareness regarding the importance of sleep health for the general public [165].
3. *Sleep apnea heterogeneity*: The current one-size-fits-all approach of diagnosing OSA patients with a sleep test then giving them CPAP has not yielded sufficient progress [166]. Improving our understanding of the varying mechanisms underlying OSA (endotypes) and the varying expression of disease (phenotypes) may be critical to approaching individualized therapy. Importantly, obesity itself is highly heterogeneous and likely contributes differently to OSA pathogenesis across patients through varying effects on upper airway anatomy, visceral and peripharyngeal adiposity, ventilatory control, inflammation, and metabolic dysfunction. Better characterization of these interactions may help identify which patients are most likely to benefit from weight loss, pharmacotherapy, PAP therapy, oral appliances, surgery, or combination approaches. Such efforts could also be helpful for OSA prevention, e.g., exercise prescriptions for patients at risk of OSA or tongue exercises for people with snoring before they develop OSA.
4. *High-throughput providers*: One idea that has been implemented in under-resourced areas to train providers to care for high volumes of patients with common conditions such as HIV and TB in South Africa. A similar approach could be applied to underserved communities to care for patients with metabolic dysfunction. Uncomplicated patients could potentially see one provider for therapy including diet, exercise, CPAP, anti-hypertensives, statins, anti-diabetic medications, and GLP-1-RA as appropriate, rather than numerous specialists. As healthcare moves from volume-based purchasing (*quantity* of care) to value-based purchasing (*quality* of care), efficient models will be required to deliver routine care such that specialists could be reserved for the more complex or refractory cases.
5. *Health equity and access*: The populations most burdened by obesity and sleep disorders—including racial/ethnic minorities and individuals with lower socioeconomic status—are often those least likely to access emerging obesity therapies [167–169]. Although incretin-based pharmacotherapies have the potential to substantially improve outcomes in obesity-related sleep disorders, high costs, insurance barriers, limited specialist access, and disparities in healthcare delivery may exacerbate existing inequities. These concerns may be particularly important given that pharmacotherapy generally requires ongoing treatment and long-term adherence to maintain benefit. It is encouraging to see that direct-to-consumer prices are gradually declining and some individuals may reduce spending on alcohol, tobacco, and groceries while receiving treatment. However, these indirect financial effects are unlikely to benefit individuals unable to afford upfront treatment. Future research should examine equitable implementation strategies, real-world access, and long-term outcomes across diverse populations, while advocacy efforts should aim to improve equitable access to evidence-based obesity and sleep care [170].
6. *Obesity has been strongly linked to cancer risk* in preclinical as well as in human studies. However, many such studies have overlooked or ignored the potential role of intermittent hypoxemia and sleep-disordered breathing in mediating this observation. Further work in this area is encouraged.

## Conclusions

Considerable progress has been made regarding our understanding of the complex interactions between sleep health and obesity. The days of providers giving patients CPAP without trying to address the underlying cause of OSA such as obesity should be over. Only by further rigorous basic and translational research as well as advocacy is major progress likely to continue.

## Data Availability

Not applicable (review).
